# Changes in mortality trends amongst common diseases during the COVID-19 pandemic in Sweden

**DOI:** 10.1177/14034948211064656

**Published:** 2021-12-21

**Authors:** Michael Axenhus, Sophia Schedin-Weiss, Bengt Winblad, Anders Wimo

**Affiliations:** 1 Division of Neurogeriatrics, Centre for Alzheimer Research, Department of Neurobiology, Care Sciences and Society, Karolinska Institutet, Sweden; 2 Theme Inflammation and Aging, Karolinska University Hospital, Sweden; 3 Primary Care, Hudiksvall-Nordanstig, Sweden

**Keywords:** Alzheimer’s disease, COVID-19, ICD-10, Parkinson’s disease, tumours, YPLL

## Abstract

**Objective:**

It has been found that COVID-19 increases deaths within common diseases in countries that have implemented strict lockdowns. In order to elucidate the proper national response to a pandemic, the mortality rates within COVID-19 and various diseases need to be studied in countries whose pandemic response differ. Sweden represents a country with lax pandemic restrictions, and we aimed to study the effects of COVID-19 on historical mortality rates within common diseases during 2020.

**Methods:**

Regression models and moving averages were used to predict expected premature mortality per the ICD-10 during 2020 using historical data sets. Predicted values were then compared to recorded premature mortality to identify changes in mortality trends.

**Results:**

Seasonal increased mortality was found within neurological diseases. Infectious diseases, tumours and cardiac disease mortality rates decreased compared to expected outcome.

**Conclusions:**

**Changes in mortality trends were observed for several common diseases during the COVID-19 pandemic. Neurological and cardiac conditions, infections and tumours are examples of diseases that were heavily affected by the pandemic. The indirect effects of COVID-19 on certain patient populations should be considered when determining pandemic impact.**

## Introduction

The public-health effects of the novel SARS-CoV-2 (COVID-19) pandemic might not be fully elucidated from public death statistics, which is the most common statistic referred to when discussing pandemic impact. Death statistics are often incomplete, as they are mainly based on the primary cause of death and exclude undocumented COVID-19 cases or deaths where COVID-19 has been a secondary cause [1]. Official COVID-19 death statistics also does not accurately reflect the indirect effects of a global pandemic, which cause huge disruptions to health-care systems. In order to determine the impact of COVID-19 on society and on health-care systems, it is important to study the mortality trends amongst vulnerable patient populations that might be affected by the pandemic.

Sweden has been a subject of debate during the 2020 COVID-19 pandemic due to its lenient pandemic response. In contrast to other Western countries, Sweden has not implemented a lockdown, nor were legal restrictions put in place [2]. The Swedish approach to the COVID-19 pandemic relied heavily on recommendations regarding social distancing and careful hygiene [3]. Although the estimate of mortality due to COVID-19 appears to be mostly correct in Sweden, the indirect effects on mortality amongst other diseases are less studied [4]. Studying the effects of the Swedish COVID-19 strategy on public-health data in the form of mortality trends within common diseases could therefore bring insight into its effectiveness and provide information to formulate future pandemic responses.

The fact that COVID-19 increases mortality amongst certain patient groups such as the frail and elderly is well known [5,6]. However, the indirect effects of COVID-19 on common diseases are less studied. Excess mortality – more deaths than would normally be expected – has been reported for several diseases during 2020 [7,8]. There have also been indications that current reporting of COVID-19 deaths is likely to underestimate the future burden on health-care systems [9,10]. Research into how common diseases have been affected during the pandemic is currently lacking and is a highly relevant area of study.

There are many reasons to assume that COVID-19 might cause increased mortality either directly or indirectly. Societal quarantine restrictions and decreased social interactions might result in increased rates of psychiatric disorders and suicides [11]. Diseases such as cardiovascular disorders and diabetes require a functional and structurally sound health-care system and are likely to have been affected because of the prioritisation of COVID-19 care [12,13]. COVID-19 deaths might also be mistaken for deaths within diseases that are identified as risk factors for COVID-19 death such as hypertension, diabetes or lung disease, especially during the first months of the pandemic, affecting data registration for such diseases [14].

The identification and characterisation of changes in the public-health burden of common diseases related to the COVID-19 pandemic are therefore important to facilitate targeted intervention amongst patients with common diseases in order to lessen the effects of this devastating pandemic, as well as to formulate future pandemic responses.

As detailed reporting on mortality amongst common diseases is lacking, with death totals focusing on COVID-19 confirmed deaths, indirect effects on mortality might be missed and the full scope of the pandemic underestimated. Using regression modelling, the amount of expected premature mortality amongst common diseases can be calculated. Derived from these data, deviations in mortality trends can be indicative of the effects of COVID-19 on certain patient categories.

As COVID-19 deaths influence health-care policy and health-care reform, we investigated years of potential life lost (YPLL) trends amongst all International Statistical Classification of Diseases and Related Health Problems – Tenth Revision (ICD-10) codes registered in Sweden during the COVID-19 pandemic. We noted several trends amongst common diseases, with temporary increased YPLL amongst neurological diseases and a decrease of YPLL within ischaemic heart disease, infectious diseases and tumours. We speculate that these trends are direct or indirect effects of COVID-19.

## Methods

### Data acquisition

Sweden is divided into 21 regions, each responsible for health care within its geographical area. The regions report independently to a central institution, the National Board of Health and Welfare, all COVID-19 and ICD-10 death causes. From these data, some epidemiological parameters such as YPLL and mortality rate are calculated and are publicly available. YPLL is calculated by subtracting the age of death from the standard year and adding the individual YPLL across each ICD-10 coded cause of death according to the following formula:

YPLL(*d,s,a,t*)=*D*(*d,s,a,t*)×*e*(*s,a,t*)

where *D*(*d,s,a,t*) is the number of deaths per cause, *d* is the cause, *s* is sex, *a* is age and *t* is time period, and *e*(*s,a,t*) is the remaining time at that sex, age and time period.

All data were obtained from the registers of the National Board of Health and Welfare and Statistics Sweden from 2010 to 2020 [15,16]. The YPLL data for all 65 ICD-10 codes according to the European shortlist of causes of death were collected and analysed. All data used were anonymised, publicly available and therefore not subject to ethical review. Data analysis was performed using IBM SPSS Statistics for Windows 28.0 (IBM Corp., Armonk, NY). All graphs were created using GraphPad Prism 9.1.1 (GraphPad Software, San Diego, CA).

### Statistics

In order to determine COVID-19 impact on vulnerable patient groups, we decided to base our study on YPLL data per ICD-10 code as described by the European shortlist of causes of death [17]. As the first wave of COVID-19 hit Sweden during the spring of 2020, we analysed data based on a six-month interval in order to determine any seasonal effects of the pandemic. The first interval of analysis was 1 January to 30 June, and the second interval was from 1 July to 31 December. We aimed to predict YPLL per ICD-10 code during 2020 using YPLL data from 2010 to 2019 to construct predicative values for common diseases during 2020. In order to identify trends amongst common disease, we used both regression modelling and moving averages. We first applied regression models to each individual ICD-10, a total of 65 codes, fitting polynomial functions and measuring the *R*^2^ value. Regression was applied in intervals of 5, 10 and 20 years, and the model with most accurate residuals was then chosen. Predicative values were calculated using function equations. The data were then validated using moving averages to forecast values for 2020. ICD-10 codes where both regression model prediction and moving averages forecast intersected were considered valid for trend prediction. Moving averages used an interval of three years over 10 years. An *R*^2^ value of at least 0.8 was used to ensure a good regression model fit. A *p*-value of <0.05 was considered significant.

We excluded small sample size ICD-10 codes, defined as an average of ⩽500 YPLL per year. The first and second half of the year from 2010 to 2019 was analysed for trends in YPLL data per ICD-10 code. Thereafter, whole-year data were analysed. The model was also applied to YPLL data for men and women separately and as a group to identify sex-specific trends. We analysed combined YPLL data for all ICD-10 codes to detect any decrease or increase in overall mortality.

We identified ICD-10 codes which fit with the mortality trend model and calculated a predicative value of YPLL per each identified ICD-10 for corresponding time periods of 2020. The predicted value was then compared to the specific registered ICD-10 YPLL value for 2020 and any significant difference detected. Using this method, it is possible to detect seasonal- and sex-specific YPLL trends within individual ICD-10 codes.

## Results

We first analysed YPLL data per ICD-10 code from 1 January to 30 June during 2010–2019. Mortality trends and good model fits were found for ICD-10 codes representing Alzheimer’s disease (AD; Supplemental Figure S1), dementia (Supplemental Figure S2), Parkinson’s disease (PD; Supplemental Figure S3), ischaemic heart disease (IHD; Supplemental Figure S4) and myocardial infarction (MI; Supplemental Figure S5). YPLL was then predicted for each identified ICD-10 code for 2020 and compared to the registered YPLL outcome. In the first six months of 2020, a significant reduction in YPLL compared to predicted outcome could be detected for IHD and MI. AD, dementia and PD, on the other hand, showed an overall increase in YPLL. Changes in PD YPLL were driven by an increase amongst men of >24% (Table I). Combined ICD-10 data for all ICD-10 codes showed more YPLL amongst men and women during the first six months of 2020, driven mostly by an increase amongst men.

**Table I.** YPLL data for the first six months of 2020.

Diagnosis	ICD-10	Sex	Expected YPLL	YPLL	YPLL difference	*p*
AD	G30	Men	4147	4709	13.55%	0.0012
		Women	8261	9014	9.12%	0.0038
		Men and women	12,408	13,162	6.08%	0.0049
Dementia	F01–F03	Men	6480	7038	8.61%	0.0091
		Women	11,972	11,902	–0.58%	0.52
		Men and women	18,069	18,940	4.82%	0.00102
PD	G20	Men	2047	2554	24.77%	0.00048
		Women	1513	1541	1.85%	0.085
		Men and women	3560	4095	15.03%	0.00064
IHD	I20–25	Men	32,057	29,104	–9.21%	0.0023
		Women	16,917	16,182	–4.34%	0.0082
		Men and women	48,974	45,288	–7.53%	0.0073
MI	I21–22	Men	14,929	12,982	–13.04%	0.0028
		Women	9043	7039	–22.16%	0.0064
		Men and women	23,972	20,021	–16.48%	0.0092
All causes of death	A00–Y89	Men	271,176	302,357	11.04%	0.0044
		Women	242,969	258,195	5.92%	0.0016
		Men and women	514,145	560,552	8.33%	0.0041

Expected YPLL compared to expected outcome show increased mortality amongst AD, dementia and PD patients. YPLL decreased amongst IHD and MI.

YPLL: years of potential life lost; AD: Alzheimer’s disease; PD: Parkinson’s disease; IHD: ischaemic heart disease; MI: myocardial infarction.

We then repeated the analysis from 1 July to 31 December during 2010–2019 to identify mortality trends for common diseases during the second half of the year. Similar to the first half of the years, changes in mortality trends were detected for AD, dementia, PD, IHD and MI. We also detected a decrease in malignant tumours amongst men and women, but we were unable to discern if this was driven by YPLL decrease amongst men or women due to limited data when divided into groups by sex (Supplemental Figure S6). IHD and MI showed a decrease in YPLL, similar to the one noted in the first six months of 2020. AD, dementia and PD showed reactionary overall decreases in YPLL. The overall YPLL for all diseases was lower during the second half of the year compared to the first half, although it was still elevated past expected levels by almost 2% (Table II).

**Table II.** YPLL data for the last six months of 2020.

Diagnosis	ICD-10	Sex	Expected YPLL	YPLL	YPLL difference	*p*
AD	G30	Men	4317	3817	–11.58%	0.00052
		Women	7832	7997	2.11%	0.0792
		Men and women	12,149	11,815	–2.75%	0.0166
Dementia	F01–F03	Men	6190	5797	–6.35%	0.0012
		Women	10,777	10,006	–7.15%	0.0078
		Men and women	16,967	15,803	–6.86%	0.0064
PD	G20	Men	2129	1963	–7.80%	0.0037
		Women	1480	1382	–6.62%	0.0091
		Men and women	3609	3345	–7.32%	0.0062
Malignant tumours	C00–97	Men and women	153,623	147,046	–4.28%	0.0083
IHD	I20–25	Men	29,880	27,130	–9.20%	0.0015
		Women	15,920	15,352	–3.57%	0.002
		Men and women	45,800	42,482	–7.24%	0.0061
MI	I21–22	Men	15,263	12,792	–16.19%	0.00059
		Women	8851	7137	–19.37%	0.00014
		Men and women	24,114	19,929	–17.36%	0.00074
All causes of death	A00–Y89	Men	266,281	270,531	1.63%	0.03716
		Women	230,351	235,720	2.37%	0.016
		Men and women	496,632	506,250	1.92%	0.034

YPLL data show a decrease amongst AD, dementia and PD patients. YPLL amongst IHD and MI continue to decrease during the second half of the year. For Malignant tumours, YPLL decreased.

Our next step was to identify yearly trends and to see if seasonal changes in YPLL affect yearly outcomes. We therefore analysed YPLL data from the whole year during the 2010–2019 period, putting the interval from 1 January to 31 December 2010–2019. Yearly mortality trends changes were identified for PD, IHD, MI, malignant tumours and lung cancer (Supplemental Figure S7). Common infectious diseases such as influenza and pneumonia also showed a whole-year decrease in all groups (Supplemental Figure S8). YPLL for IHD and MI was significantly decreased amongst both men and women during 2020, with men and women showing a combined YPLL decrease of 4% and 17%, respectively, compared to predicted outcome. PD YPLL decreased by 7.5% during 2020. Interestingly, whole-year analysis showed no significant difference in outcome compared to expected YPLL for AD and dementia, indicating no yearly change. Combined YPLL loss for the whole year showed an increased overall mortality in Sweden during 2020 of >5% (Table III).

**Table III.** Whole-year YPLL data for 2020.

Diagnosis	ICD-10	Sex	Expected YPLL	YPLL	YPLL difference	*p*
AD	G30	Men	8464	8089	–4.43%	0.0067
		Women	16,096	16,479	2.38%	0.074
		Men and women	24,569	24,559	–0.04%	0.85
Dementia	F01–F03	Men	12,658	12,835	1.40%	0.41
		Women	12,197	12,512	2.58%	0.34
		Men and women	24,855	25,347	1.98%	0.31
PD	G20	Men	4176	4517	8.17%	0.0014
		Women	8670	9538	10.01%	0.0057
		Men and women	12,846	14,055	9.41%	0.0031
Malignant tumours	C00–97	Men and Women	304,319	296,353	–2.62%	0.0024
Lung cancer	C33–34	Men and women	51,082	47,783	–6.46%	0.0038
IHD	I20–25	Men	60,941	57,417	–5.78%	0.0077
		Women	32,600	31,898	–2.15%	0.0034
		Men and women	93,541	89,315	–4.52%	0.00822
MI	I21–22	Men	31,773	25,774	–18.88%	0.00034
		Women	17,910	15,253	–14.84%	0.00055
		Men and women	49,683	41,027	–17.42%	0.00022
Influenza	J9–11	Men	1825	811	–55.56%	0.0000054
		Women	4324	2098	–51.48%	0.00009822
		Men and women	6149	2909	–52.69%	0.0007822
Pneumonia	J12–18	Men	7307	5688	–22.16%	0.00033
		Women	12,077	10,072	–16.60%	0.00045
		Men and women	19,384	15,760	–18.70%	0.00029
All causes of death	A00–Y89	Men	537,457	572,888	6.19%	0.026
		Women	473,320	493,915	4.22%	0.016
		Men and women	1,010,777	1,066,802	5.32%	0.0013

AD and dementia showed no yearly difference compared to previous years. IHD and MI decreased during the whole year, with the largest decrease seen in men. Lung cancer and malignant tumours were both significant decreased on a yearly basis. PD showed a large decrease in whole-year YPLL. Influenza and pneumonia showed large decreases in YPLL.

## Discussion

We built statistical models to notice trends in premature mortality, measured as YPLL, per ICD-10 code during the COVID-19 pandemic in Sweden in order to elucidate the pandemic’s effect on public health during 2020. We compared expected YPLL outcome to recorded outcome per ICD-10 code and found mortality trends within neurological, cardiological, infectious and malignant diseases. Premature mortality within neurological debilitating diseases such as AD, dementia and PD increased during the first six months of 2020 and decreased during the last six months of 2020. YPLL data for malignant diseases decreased on a whole-year basis for 2020. IHD and MI YPLL decreased during 2020 in both the first and second halves of the year. YPLL loss assigned to influenzas and pneumonia also decreased during 2020.

COVID-19 has placed a huge strain on both health-care systems and caregivers across the world. Thus, a trend change in mortality within common diseases is not unexpected, and our data display that these effects might be larger than previously anticipated.

We found a larger than expected decrease in IHD and MI during both halves of 2020. Cardiovascular incidents are common causes of deaths, and cardiovascular care is highly dependent on a specialised and well-developed health-care systems, which are disrupted during pandemic conditions. We also noticed a mortality decrease in malignant tumours. Malignant tumours require careful diagnostics and often need specialised treatment. A decrease in cancer care was reported in Sweden during 2020. Therefore, decreased cancer diagnosis and treatment coupled with quarantine restrictions and unwillingness to seek health care during the pandemic could be factors that have influenced this documented decrease in cancer mortality. Neurological diseases with a high burden of care such as AD, dementia and PD reported a mortality increase during the first half of 2020 and a mortality decrease during the second half of the year. AD, dementia and PD patients are often, particularly in the end-stage of the disease, cared for in nursing homes. Nursing homes in Sweden were some of the hardest hit facilities during 2020, with a significant portion of COVID-19 deaths during the first half of 2020 represented by nursing home occupants [18]. When analysing combined YPLL data for all ICD-10 codes during 2020, an increase of roughly 5% was observed, indicating an increased overall mortality in Sweden during 2020, particularly during the first half of the year.

### Cardiovascular disease

We found mortality changes affecting IHD, defined as ICD-10 coded I20–25. We also detected trend changes in the ICD-10 group I21–22, representing MI. IHD and MI mortality decreased substantially during 2020 in both the first and second halves of the year. Whole-year data showed a total decrease in IHD mortality as large as 4.5% and an MI mortality decrease of 17%. This trend was mostly driven by changes amongst men. IHD and MI patients are large patient groups, and their YPLL data indicate a significant disturbance to cardiovascular care. Since there was a uniform decrease during the year, it is likely that the explanation to the decrease in YPLL is multifactorial. Decreased compliance with fewer doctors’ visits due to quarantine measurements, the prioritisation of COVID-19 related care and a decrease in the number of diagnoses are factors that could influence YPLL data [19,20]. Similar trends have been noted in other countries that have shown a decrease in hospitalisation and percutaneous coronary interventions during the COVID-19 pandemic, likely resulting in decreased registered mortality due to cardiac events [21–24]. It is clear that patients with cardiac conditions were affected by the COVID-19 pandemic, and disease mortality and burden might not be accurately represented by official YPLL statistics.

### Neurological diseases

Our data showed an increase in premature mortality amongst AD, dementia and PD patients during the first half year of 2020, with a reactionary decrease in mortality during the second half of the year. Whole-year results showed an increase in mortality amongst PD and no difference in AD and dementia mortality. This result indicates a substantial effect of the spring wave of COVID-19 during 2020 on the AD, dementia and PD patient population. It is not unexpected to find higher mortality amongst AD and dementia patients. However, most reports do not show a seasonal trend like Sweden but rather an overall increase in mortality. In the USA, for example, AD mortality rose almost 16% during 2020 according to official statistics [25]. This is most likely due to variations in time and intensity of COVID-19 waves between different countries. There have also been indications of decreases in AD diagnoses. Public data from the National Health Service in the UK has demonstrated a significant decrease in dementia diagnoses [26]. Both of these factors, if true in Sweden, could explain the seasonal YPLL loss noted in AD and dementia due to increased mortality and decreased diagnoses. Several studies have also identified a decrease in stroke admissions and mortality, although we were unable to detect such a trend in our study due to large yearly variations in stroke YPLL data [27,28].

Increased overall mortality amongst PD patients has been noted in other countries. Recent studies have shown that PD is associated with higher risk of both severe COVID-19 infection and death [29,30]. The pandemic has also caused disruptions to the daily lives of PD patients, with many reporting increases in symptoms and decreased medical access, prompting potential complications [31]. A substantial number of the most vulnerable AD, dementia and PD patients can be found within nursing care homes, which is also where most of them die [32]. A study into Swedish long-term care facilities found an increase in mortality amongst residents, with neurological diseases being a risk factor [33]. Pandemic conditions obviously make it difficult to sustain the high level of care that nursing home residents often require [34]. Nursing care homes deaths present a significant portion of all COVID-19 deaths in Sweden, and rate of infections amongst staff and patients has been high [35,36]. In some cases, a third of nursing home residents died during the pandemic [37]. Discussions have been raised regarding the best care for nursing home residents, and investigations into nursing home care has been enacted [38,39].

### Tumours

Tumour diagnostics have advanced significantly during the last decades. This has enabled more accurate diagnoses and treatment, with a subsequent increase in deaths for this patient group, although a lower overall mortality. Today, tumours still represent a significant portion of total deaths in Sweden. We found whole-year decreases of YPLL related to lung cancer and malignant tumours. The COVID-19 pandemic limited screening and referral rates, which has caused a temporary decrease in common cancers [40–42]. Our study found YPLL loss amongst both lung cancer and malignant tumours, which could be indicative of decreased oncological care with fewer cancer diagnoses and treatments. Although we detected an overall decrease in YPLL amongst malignant tumours, not all of that change can be explained by the decrease in lung cancers, meaning that YPLL of other malignancies are probably affected. ICD-10 codes for malignancies are often specialised and comprise only a few YPLL per code, making it difficult to predict trends based on public data.

Internationally, oncology has been one of the health-care areas hit the hardest during the pandemic, with delays in diagnosis and treatment [43]. Sweden has been no exception, with heavily reduced diagnoses and, as a consequence, treatment rates [44]. A decrease in tumour diagnoses and subsequent treatment could explain a smaller than expected YPLL amongst patients with lung cancer and other malignant tumours during 2020. If these assumptions are accurate, then subsequent years could display a large increase in both tumour diagnostics and treatments as health-care systems regain effectiveness.

### Infectious diseases

Whole-year YPLL data showed marked decreases amongst influenza and pneumonia groups. Influenza decreased by almost half, and pneumonia decreased by 18%. Both pneumonia and influenza are seasonal and can be prevented effectively with restrictions and decreased social contact – common tactics during the COVID-19 pandemic in Sweden. These results correlate well with studies in other countries, which have found that restrictions and quarantines reduced the spread of common infectious diseases such as influenza and pneumonia [45–47]. However, countries which employed stricter quarantine and pandemic regulations had an even bigger drop in influenza rates than those in Sweden [45]. Some of this change could also be explained by patients in groups commonly affected by influenza and pneumonia being particular at risk of COVID-19 infection, such as the old and frail. If COVID-19 has a more efficient community spread than pneumonia and influenza, suppression of these diseases amongst certain populations could be a contributing factor to lower YPLL amongst these diseases [48,49]. This has also raised concern over whether the suppression of common infectious diseases because of COVID-19 might drive the development of more efficient and contagious strains [50].

### Weaknesses

Many ICD-10 codes did not show a good fit for regression modelling. This was due to the nature of the data, with either a low number of cases or irregularities in mortality between years, likely explained by changes in diagnoses, seasonality or treatment methods. We were only able to identify trends in ICD-10 codes that represent common diseases and that have established diagnostic and treatment methods, such as ischaemic heart conditions, infections, oncological and neurological diseases. Smaller trends amongst uncommon diseases might go unnoticed with this approach and would require more detailed studies.

## Conclusions

Mortality trends within common diseases were affected during the COVID-19 pandemic in Sweden. There are signs of both overall trends, with general decreased mortality within cardiovascular diseases, infections and tumours, as well as more acute affects in the form of temporary increased mortality within some neurological diseases.

Patients within these disease categories are more susceptible to COVID-19 infections either directly due to risk factors or indirectly due to the pandemic’s effect on society and health-care systems. This should be taken into account when discussing pandemic impact.

Our data suggest that the effects of COVID-19 on public health might be greater than previously expected, and precautions should be taken to account for these changes as health systems recover.

## Supplemental Material

sj-jpg-1-sjp-10.1177_14034948211064656 – Supplemental material for Changes in mortality trends amongst common diseases during the COVID-19 pandemic in Sweden Click here for additional data file. Supplemental material, sj-jpg-1-sjp-10.1177_14034948211064656 for Changes in mortality trends amongst common diseases during the COVID-19 pandemic in Sweden by Michael Axenhus, Sophia Schedin-Weiss, Bengt Winblad and Anders Wimo in Scandinavian Journal of Public Health

sj-jpg-2-sjp-10.1177_14034948211064656 – Supplemental material for Changes in mortality trends amongst common diseases during the COVID-19 pandemic in Sweden Click here for additional data file. Supplemental material, sj-jpg-2-sjp-10.1177_14034948211064656 for Changes in mortality trends amongst common diseases during the COVID-19 pandemic in Sweden by Michael Axenhus, Sophia Schedin-Weiss, Bengt Winblad and Anders Wimo in Scandinavian Journal of Public Health

sj-jpg-3-sjp-10.1177_14034948211064656 – Supplemental material for Changes in mortality trends amongst common diseases during the COVID-19 pandemic in Sweden Click here for additional data file. Supplemental material, sj-jpg-3-sjp-10.1177_14034948211064656 for Changes in mortality trends amongst common diseases during the COVID-19 pandemic in Sweden by Michael Axenhus, Sophia Schedin-Weiss, Bengt Winblad and Anders Wimo in Scandinavian Journal of Public Health

sj-jpg-4-sjp-10.1177_14034948211064656 – Supplemental material for Changes in mortality trends amongst common diseases during the COVID-19 pandemic in Sweden Click here for additional data file. Supplemental material, sj-jpg-4-sjp-10.1177_14034948211064656 for Changes in mortality trends amongst common diseases during the COVID-19 pandemic in Sweden by Michael Axenhus, Sophia Schedin-Weiss, Bengt Winblad and Anders Wimo in Scandinavian Journal of Public Health

sj-jpg-5-sjp-10.1177_14034948211064656 – Supplemental material for Changes in mortality trends amongst common diseases during the COVID-19 pandemic in Sweden Click here for additional data file. Supplemental material, sj-jpg-5-sjp-10.1177_14034948211064656 for Changes in mortality trends amongst common diseases during the COVID-19 pandemic in Sweden by Michael Axenhus, Sophia Schedin-Weiss, Bengt Winblad and Anders Wimo in Scandinavian Journal of Public Health

sj-jpg-6-sjp-10.1177_14034948211064656 – Supplemental material for Changes in mortality trends amongst common diseases during the COVID-19 pandemic in Sweden Click here for additional data file. Supplemental material, sj-jpg-6-sjp-10.1177_14034948211064656 for Changes in mortality trends amongst common diseases during the COVID-19 pandemic in Sweden by Michael Axenhus, Sophia Schedin-Weiss, Bengt Winblad and Anders Wimo in Scandinavian Journal of Public Health

sj-jpg-7-sjp-10.1177_14034948211064656 – Supplemental material for Changes in mortality trends amongst common diseases during the COVID-19 pandemic in Sweden Click here for additional data file. Supplemental material, sj-jpg-7-sjp-10.1177_14034948211064656 for Changes in mortality trends amongst common diseases during the COVID-19 pandemic in Sweden by Michael Axenhus, Sophia Schedin-Weiss, Bengt Winblad and Anders Wimo in Scandinavian Journal of Public Health

sj-jpg-8-sjp-10.1177_14034948211064656 – Supplemental material for Changes in mortality trends amongst common diseases during the COVID-19 pandemic in Sweden Click here for additional data file. Supplemental material, sj-jpg-8-sjp-10.1177_14034948211064656 for Changes in mortality trends amongst common diseases during the COVID-19 pandemic in Sweden by Michael Axenhus, Sophia Schedin-Weiss, Bengt Winblad and Anders Wimo in Scandinavian Journal of Public Health
