# Supplementary figures and images for: Changes in mortality trends amongst common diseases during the COVID-19 pandemic in Sweden
Source: Scand J Public Health. 2021 Dec 21;50(6):748–55. doi: 10.1177/14034948211064656 (PMC9361422; doi:10.1177/14034948211064656)

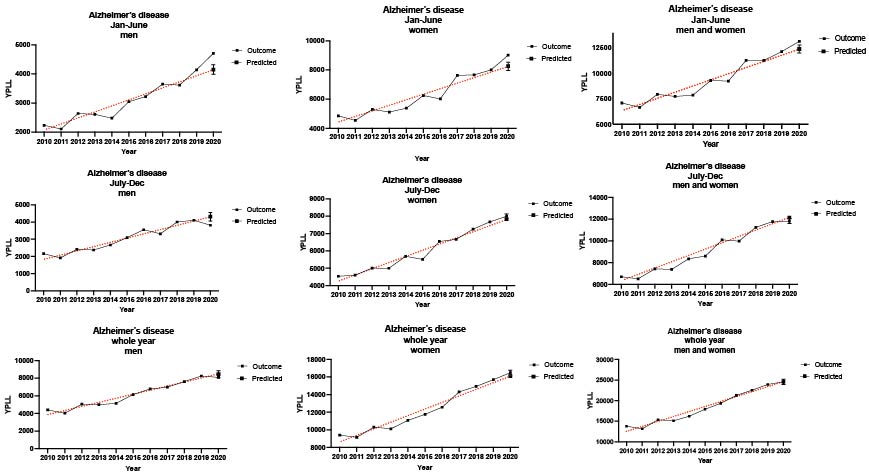

Supplement: sj-jpg-1-sjp-10.1177_14034948211064656 – Supplemental material for Changes in mortality trends amongst common diseases during the COVID-19 pandemic in Sweden [file sj-jpg-1-sjp-10.1177_14034948211064656.jpg]

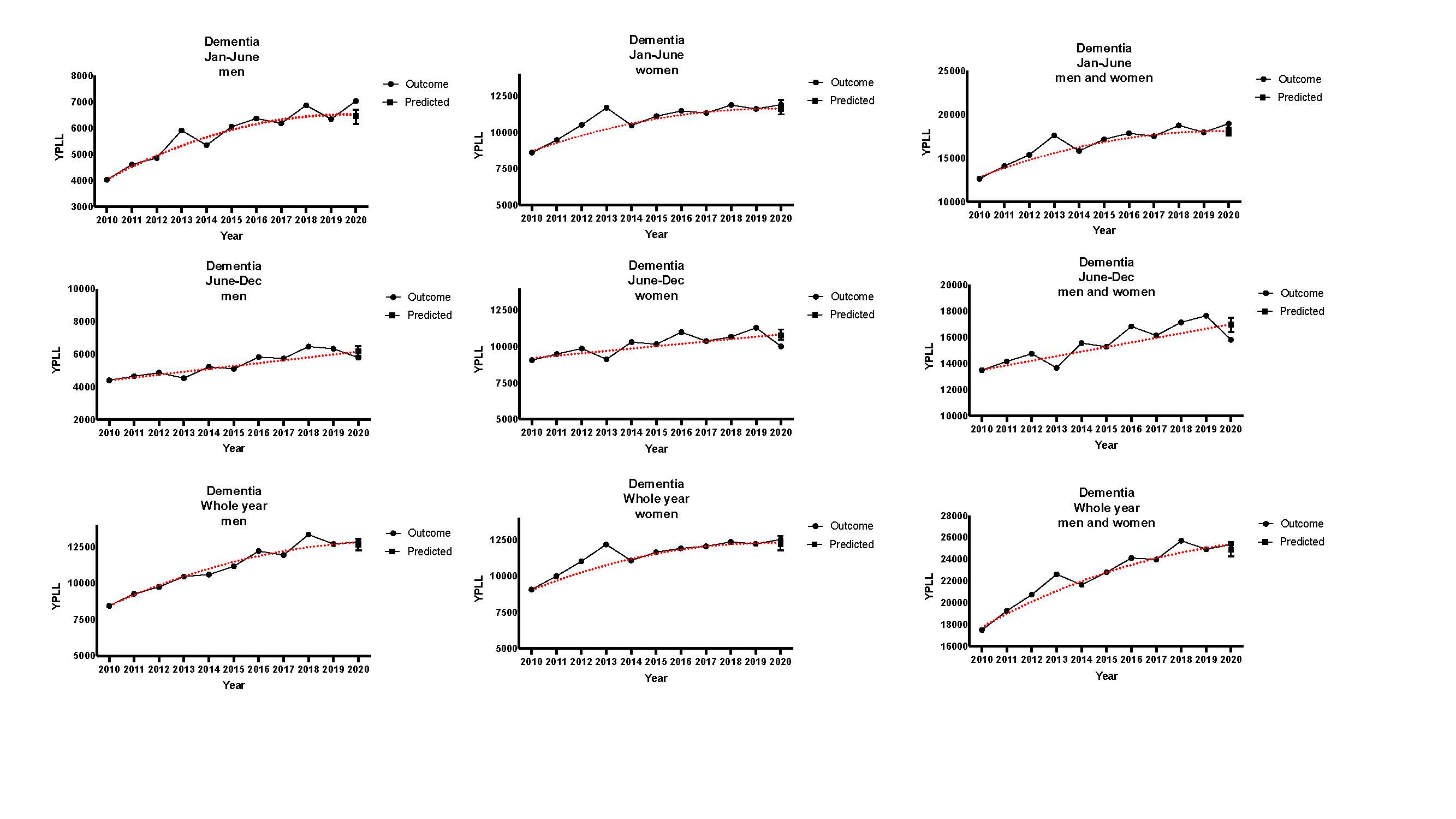

Supplement: sj-jpg-2-sjp-10.1177_14034948211064656 – Supplemental material for Changes in mortality trends amongst common diseases during the COVID-19 pandemic in Sweden [file sj-jpg-2-sjp-10.1177_14034948211064656.jpg]

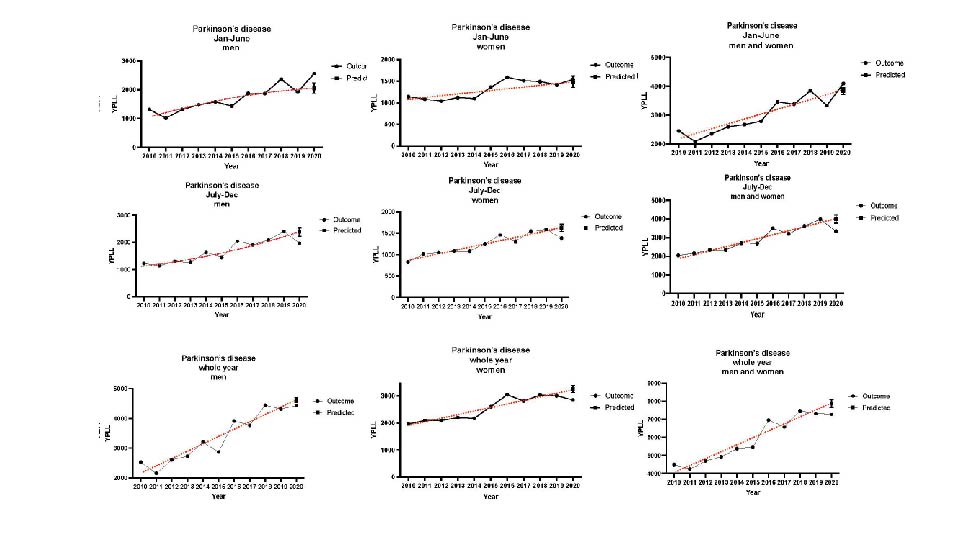

Supplement: sj-jpg-3-sjp-10.1177_14034948211064656 – Supplemental material for Changes in mortality trends amongst common diseases during the COVID-19 pandemic in Sweden [file sj-jpg-3-sjp-10.1177_14034948211064656.jpg]

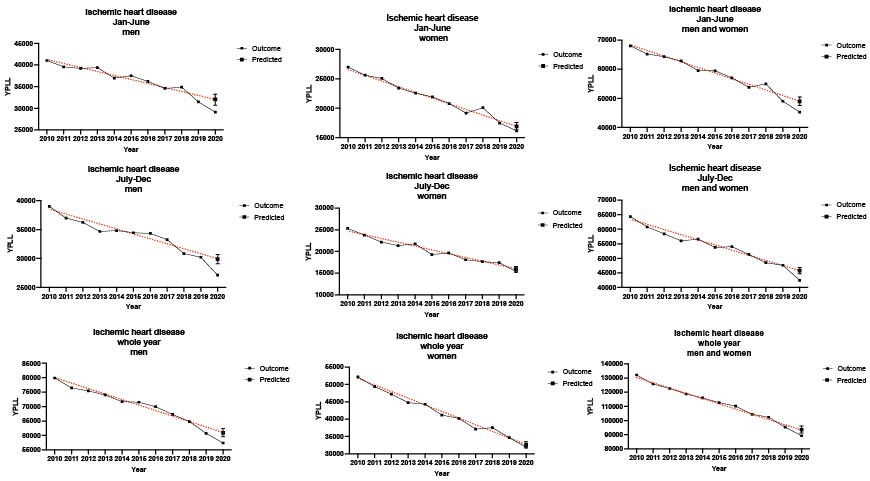

Supplement: sj-jpg-4-sjp-10.1177_14034948211064656 – Supplemental material for Changes in mortality trends amongst common diseases during the COVID-19 pandemic in Sweden [file sj-jpg-4-sjp-10.1177_14034948211064656.jpg]

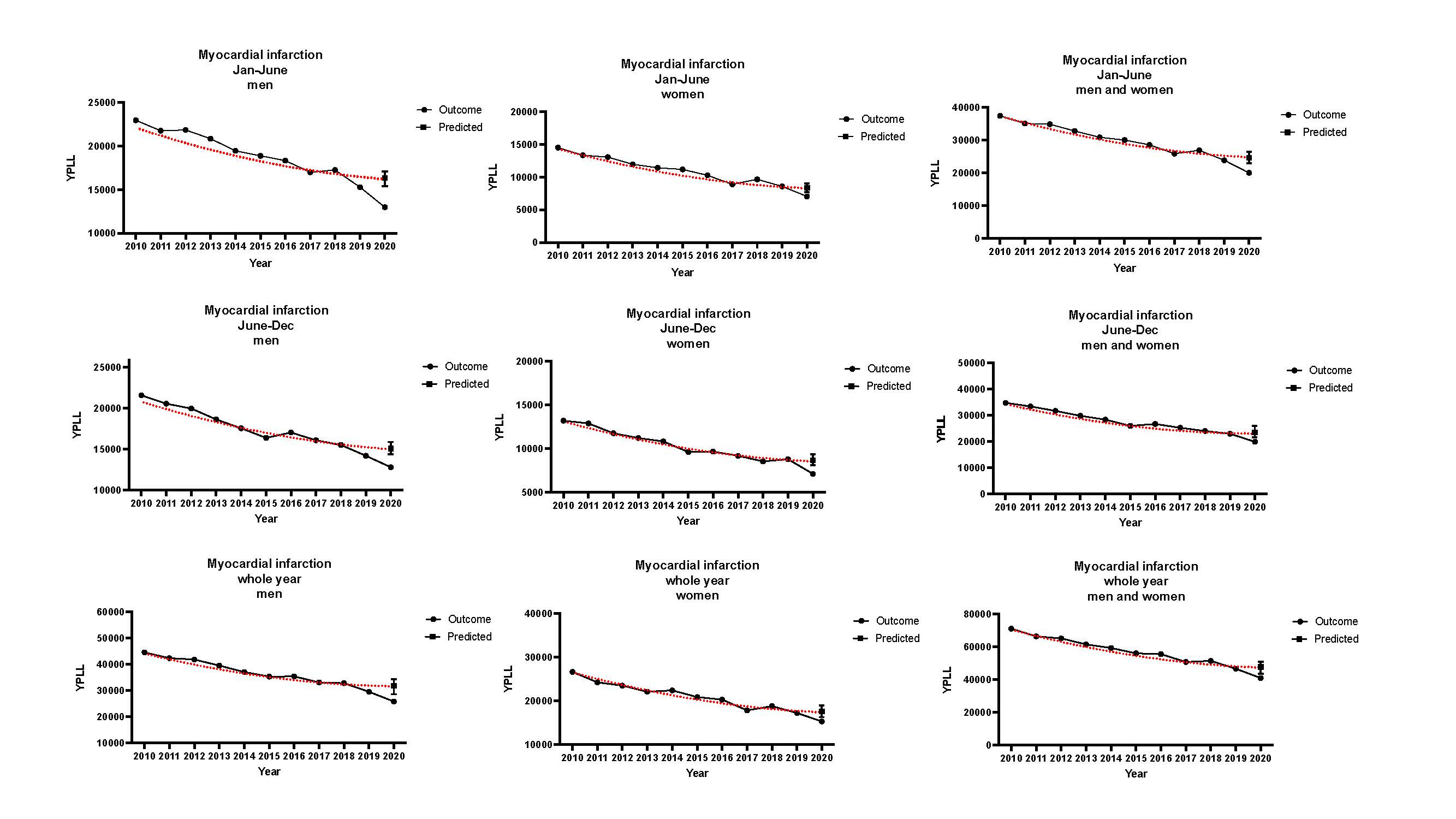

Supplement: sj-jpg-5-sjp-10.1177_14034948211064656 – Supplemental material for Changes in mortality trends amongst common diseases during the COVID-19 pandemic in Sweden [file sj-jpg-5-sjp-10.1177_14034948211064656.jpg]

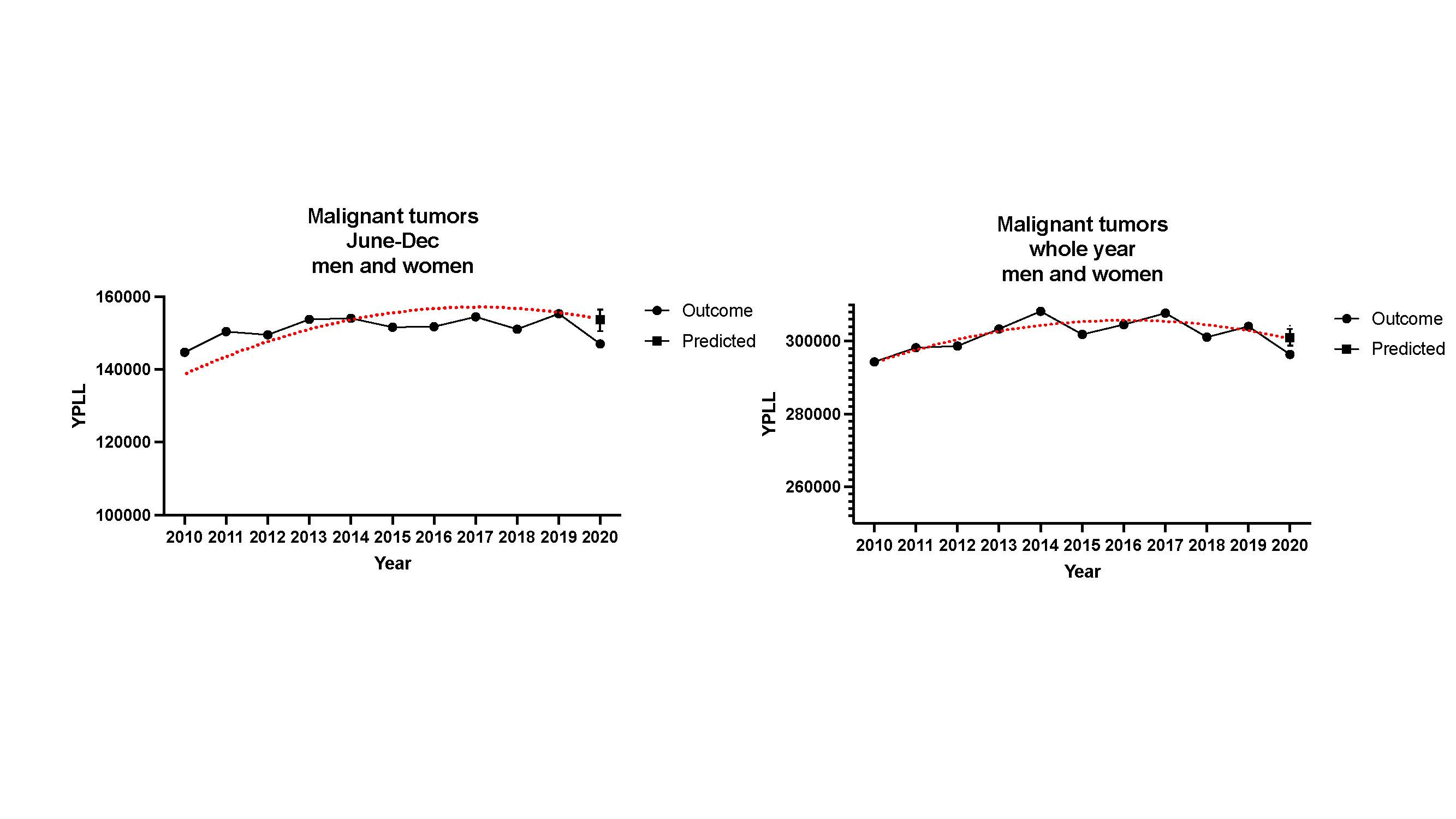

Supplement: sj-jpg-6-sjp-10.1177_14034948211064656 – Supplemental material for Changes in mortality trends amongst common diseases during the COVID-19 pandemic in Sweden [file sj-jpg-6-sjp-10.1177_14034948211064656.jpg]

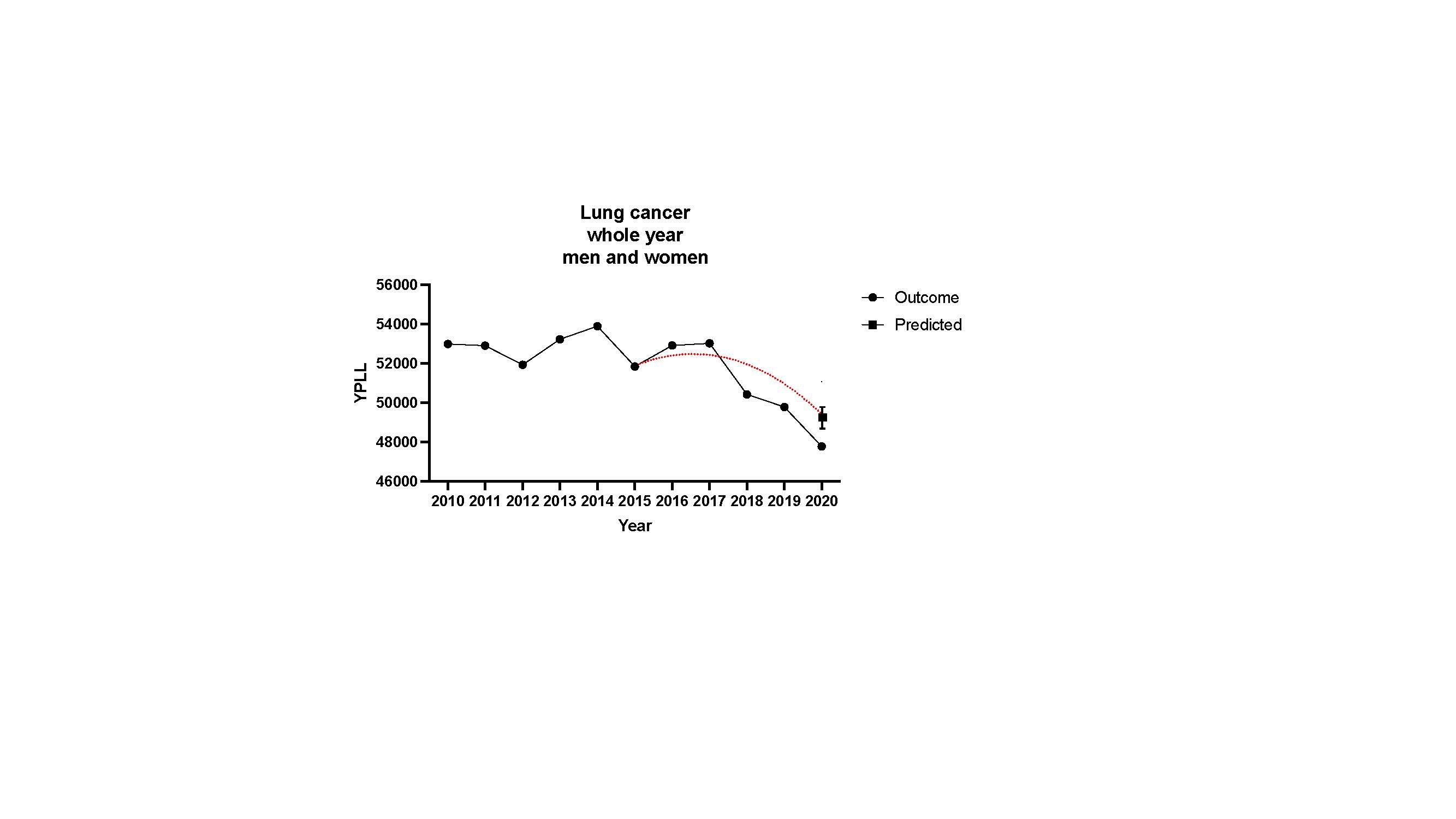

Supplement: sj-jpg-7-sjp-10.1177_14034948211064656 – Supplemental material for Changes in mortality trends amongst common diseases during the COVID-19 pandemic in Sweden [file sj-jpg-7-sjp-10.1177_14034948211064656.jpg]

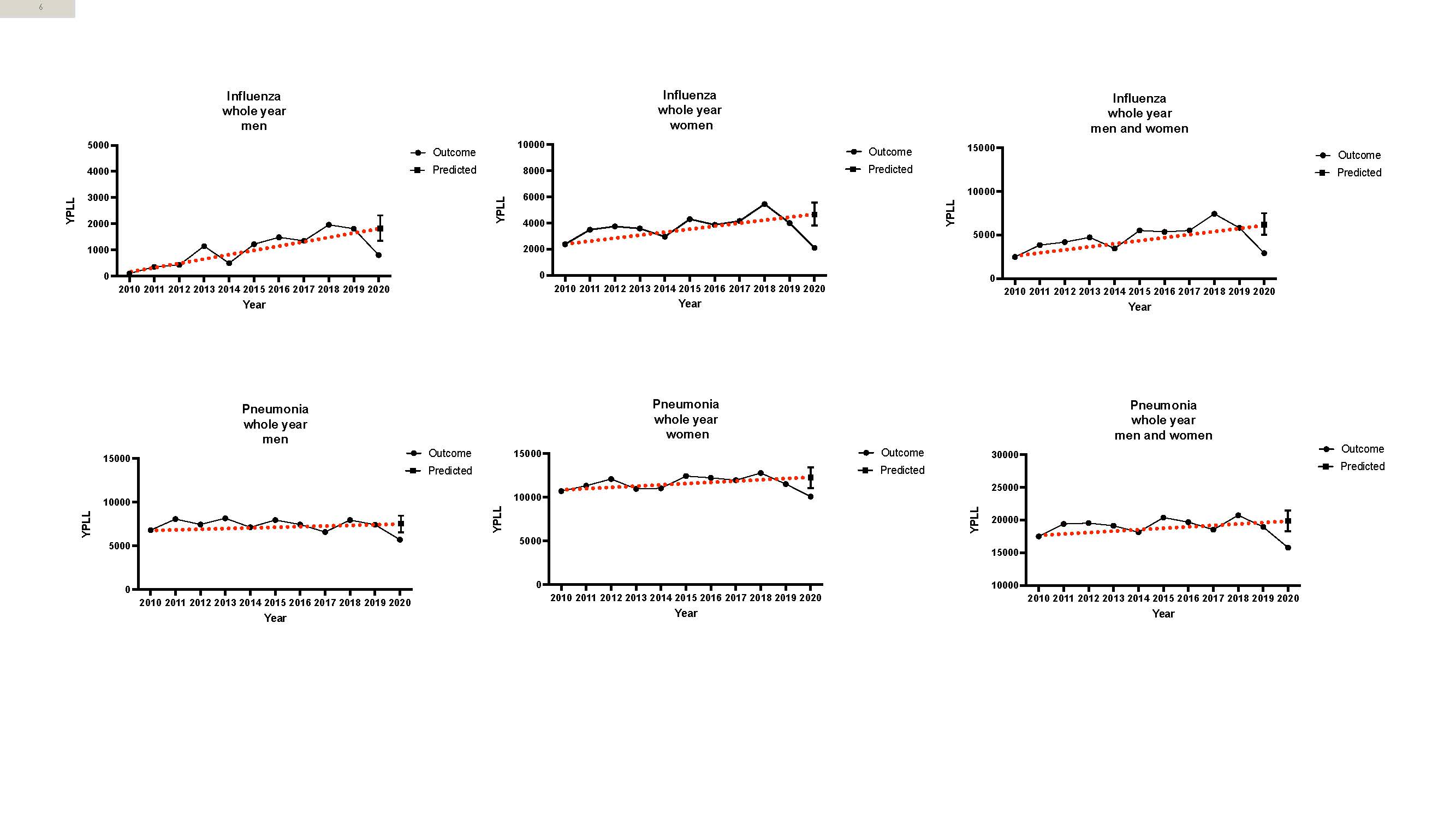

Supplement: sj-jpg-8-sjp-10.1177_14034948211064656 – Supplemental material for Changes in mortality trends amongst common diseases during the COVID-19 pandemic in Sweden [file sj-jpg-8-sjp-10.1177_14034948211064656.jpg]
